# The *O*-mannosylation and production of recombinant APA (45/47 KDa) protein from *Mycobacterium tuberculosis *in *Streptomyces lividans *is affected by culture conditions in shake flasks

**DOI:** 10.1186/1475-2859-10-110

**Published:** 2011-12-20

**Authors:** Ramsés A Gamboa-Suasnavart, Norma A Valdez-Cruz, Laura E Cordova-Dávalos, José A Martínez-Sotelo, Luis Servín-González, Clara Espitia, Mauricio A Trujillo-Roldán

**Affiliations:** 1 Unidad de Bioprocesos, Instituto de Investigaciones Biomédicas, Universidad Nacional Autónoma de México. AP. 70228, México, D.F., CP. 04510, México; 2 Departamento de Biología Molecular y Biotecnología, Instituto de Investigaciones Biomédicas, Universidad Nacional Autónoma de México, AP. 70228, México, D.F., CP. 04510, México; 3 Departamento de Inmunología, Instituto de Investigaciones Biomédicas, Universidad Nacional Autónoma de México. AP. 70228, México, D.F., CP. 04510, México

**Keywords:** Shake flasks, shear, oxygenation, APA 45/47 kDa, O-mannosylation, Mycobacterium tuberculosis, Streptomyces lividans

## Abstract

**Background:**

The Ala-Pro-rich *O*-glycoprotein known as the 45/47 kDa or APA antigen from *Mycobacterium tuberculosis *is an immunodominant adhesin restricted to mycobacterium genus and has been proposed as an alternative candidate to generate a new vaccine against tuberculosis or for diagnosis kits. In this work, the recombinant *O*-glycoprotein APA was produced by the non-pathogenic filamentous bacteria *Streptomyces lividans*, evaluating three different culture conditions. This strain is known for its ability to produce heterologous proteins in a shorter time compared to *M. tuberculosis*.

**Results:**

Three different shake flask geometries were used to provide different shear and oxygenation conditions; and the impact of those conditions on the morphology of *S. lividans *and the production of rAPA was characterized and evaluated. Small unbranched free filaments and mycelial clumps were found in baffled and coiled shake flasks, but one order of magnitude larger pellets were found in conventional shake flasks. The production of rAPA is around 3 times higher in small mycelia than in larger pellets, most probably due to difficulties in mass transfer inside pellets. Moreover, there are four putative sites of *O*-mannosylation in native APA, one of which is located at the carboxy-terminal region. The carbohydrate composition of this site was determined for rAPA by mass spectrometry analysis, and was found to contain different glycoforms depending on culture conditions. Up to two mannoses residues were found in cultures carried out in conventional shake flasks, and up to five mannoses residues were determined in coiled and baffled shake flasks.

**Conclusions:**

The shear and/or oxygenation parameters determine the bacterial morphology, the productivity, and the *O*-mannosylation of rAPA in *S. lividans*. As demonstrated here, culture conditions have to be carefully controlled in order to obtain recombinant *O*-glycosylated proteins with similar "quality" in bacteria, particularly, if the protein activity depends on the glycosylation pattern. Furthermore, it will be an interesting exercise to determine the effect of shear and oxygen in shake flasks, to obtain evidences that may be useful in scaling-up these processes to bioreactors. Another approach will be using lab-scale bioreactors under well-controlled conditions, and study the impact of those on rAPA productivity and quality.

## Background

*Mycobacterium tuberculosis *is the main bacterial pathogen causing latent infection in more than a third of the world population, and is also developing resistance to virtually every new drug used to treat tuberculosis, resulting recently in a global emergence of extensively drug-resistant tuberculosis [1-3]. At least 41 glycoproteins were identified in culture filtrates of *M. tuberculosis, *and most of them seem to play important roles in *M. tuberculosis *infection and host immune response [4-6]. Therefore, some of those glycosylated antigens are good candidates for vaccine development and improvement of diagnosis [6-9]. The first described mannosylated protein in Actinomycetes was the Ala-Pro-rich Antigen (also known as 45/47 kDa or APA protein) in *M. tuberculosis*, which is immunodominant [10-12]. The specific role of APA is not clearly defined; however, it is reported as an adhesin [13], as well as an antigen elicitor of lympho-proliferative responses and cytokine production [8]. It is worth of note, that the T cell response induced by APA is dependent of its glycosylation status specifically of the *O*-mannosylation of four of their threonines [14]. Particularly, Thr277 presents heterogeneity from none to three mannose units in native APA [11]. In addition, none to four mannose units were found in Thr277 of the rAPA produced in *S. lividans *[15].

Strains of *Streptomyces sp. *are well known as good producers of extracellular recombinant proteins; these strains also have the ability to glycosylate their own proteins, as well as heterologous proteins [15-22]. Recent studies in genetics and cell biology have revealed analogies between *Streptomyces sp. *and *Mycobacterium sp.*, both belonging to the phylum Actinobacteria [23]. Moreover, *S. lividans *allows the production, both in shake flasks [15] and in bioreactor [24], of large amounts of glycosylated rAPA suitable for biochemical studies and immunological assays. However, no detailed study of the effect of culture conditions on the productivity and *O*-glycosylation of rAPA has been reported.

On the other hand, probably more than 90% of submerged cultures for microbial research purposes are performed in shake flasks [25,26]. These studies are carried out due to their low cost, simplicity of operation and availability to do many experiments simultaneously [27,28]. Almost all the tasks, such as screening of strains, media optimization, strain development, elucidation of metabolic pathways, investigations of process conditions, and evaluation of fundamental growth kinetics are made in these vessels [27]. In shake flasks the momentum and heat transfer is influenced by the geometry of the rotating bulk liquid, that is the contact area between the liquid and the friction area, *i.e. *the flask inner wall (and indentations in baffled flasks, or the coiled stainless steel spring in flasks) [29]. On the other hand, the mass transfer (mainly oxygen) is influenced by the wet wall exposed to the surrounding air, this is the mass exchange area [29]. Modifications of shake flasks by the introduction of baffles and other enhancements (like stainless steel spring coils) are frequently necessary in order to provide sufficient aeration and shear stress [25,29,30]. In *S. lividans *cultures, coiled and baffled flasks are normally used [30-33], but no detailed study has been done on how the geometry of the flasks can affect recombinant protein production, and its *O*-glycosylation as a function of the morphology of the filamentous bacteria.

**Figure 1:**
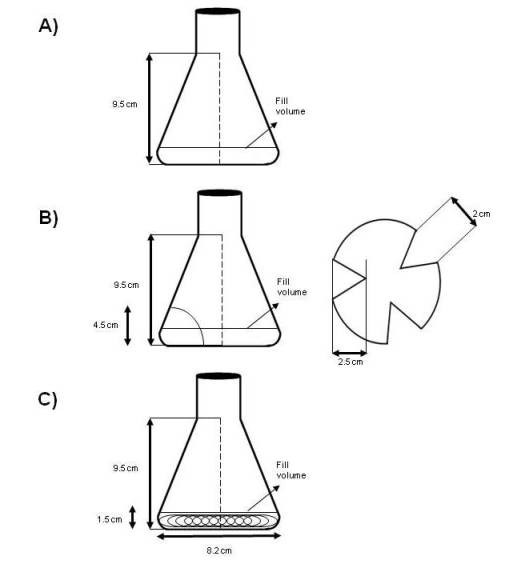
**Schematic diagrams of shake flasks used: A. Conventional Normal (NF); B. Baffled (BF); and C. Coiled shake Flasks (CF)**.

The purpose of this study was to evaluate the culture of *S. lividans *under three different shake flask geometries (conventional Erlenmeyer, baffled and coiled shake flasks, as shown in Figure 1), given different shear and oxygenation conditions, on the morphology of filamentous bacteria, their growth kinetics, and rAPA production and carbohydrate composition at the C-terminus. Furthermore, to our knowledge there are no reports regarding the influence of culture conditions on protein *O*-glycosylation by bacteria. However, there are some reports indicating modifications in the *N*-glycosylation pattern in recombinant proteins produced by eukaryotic cells due to variations in culture conditions [34-37].

## Results and Discussion

**Figure 2:**
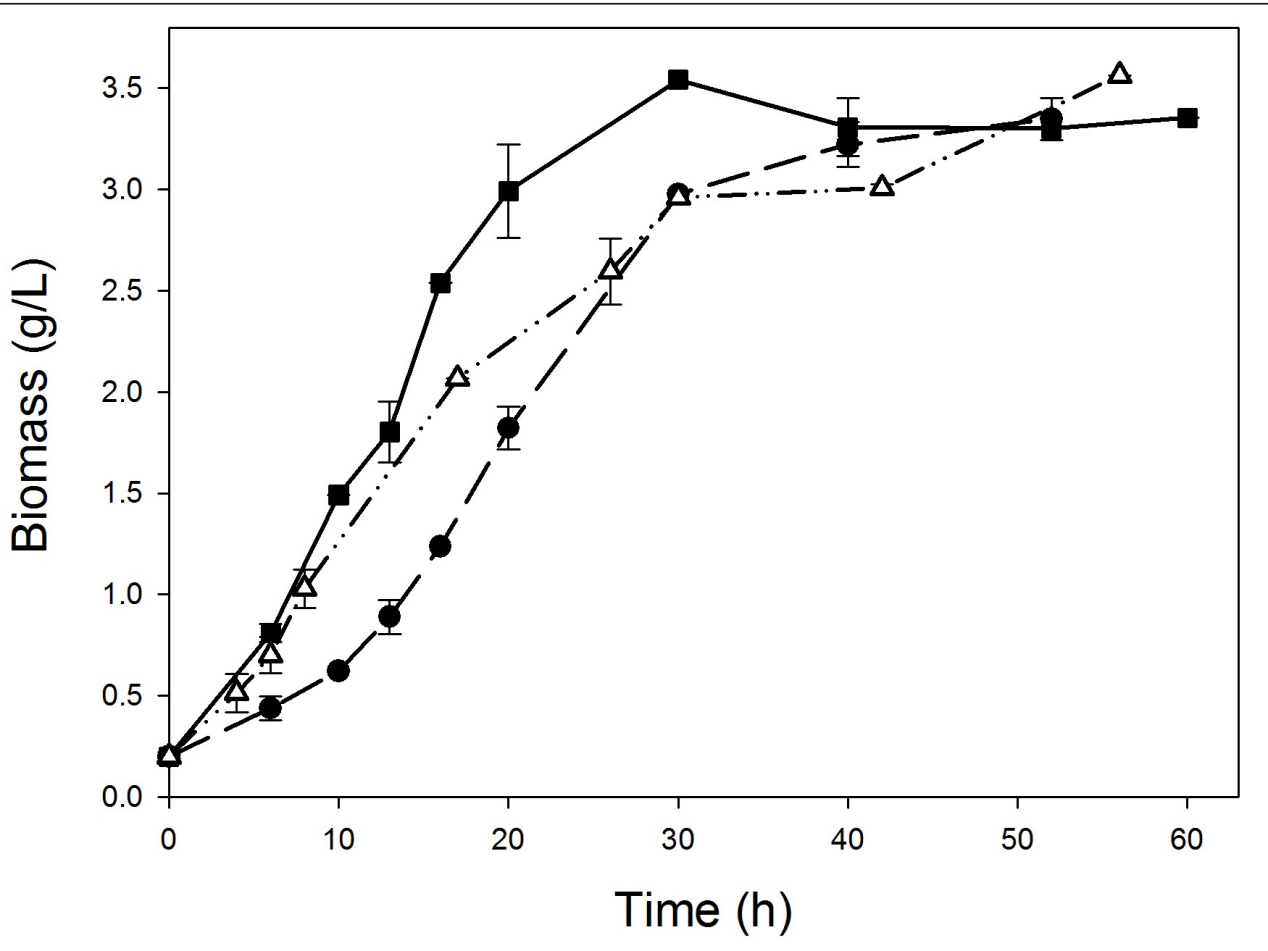
**Kinetics of biomass growth of *S. lividans *producing rAPA from *M. tuberculosis*, in conventional (squares); baffled (triangles); and coiled (dots) shake flasks**.

The effect of three different shear and oxygenation conditions using conventional, baffled and coiled shake flasks (Figure 1) on *S. lividans *growth, morphology, recombinant APA protein production and *O*-glycosylation was evaluated. Kinetics of bacterial growth is shown in Figure 2. The final biomass concentration was not affected significantly, being around 3.3 g/L dry weight, specifically, 3.5 ± 0.2 g/L for coiled flasks and 3.3 ± 0.1 g/L for conventional and baffled flasks. Moreover, the specific growth rate was not significantly different by Tukey HSD Test (p = 0.05): in coiled shake flasks 0.12 ± 0.02 h^-1^, in baffled flasks 0.11 ± 0.01 h^-1^; and in conventional flasks 0.10 ± 0.02 h^-1^. Until differences in specific growth rates between baffled (or coiled), and conventional flasks were reported, a lag phase in conventional and baffled flasks were observed and comparing only the growth slopes, similar tendencies can be found [38]. A similar lag phase were observed in this work (figure 1), surely due to a germination of spores delay in conventional shake flasks.

**Figure 3:**
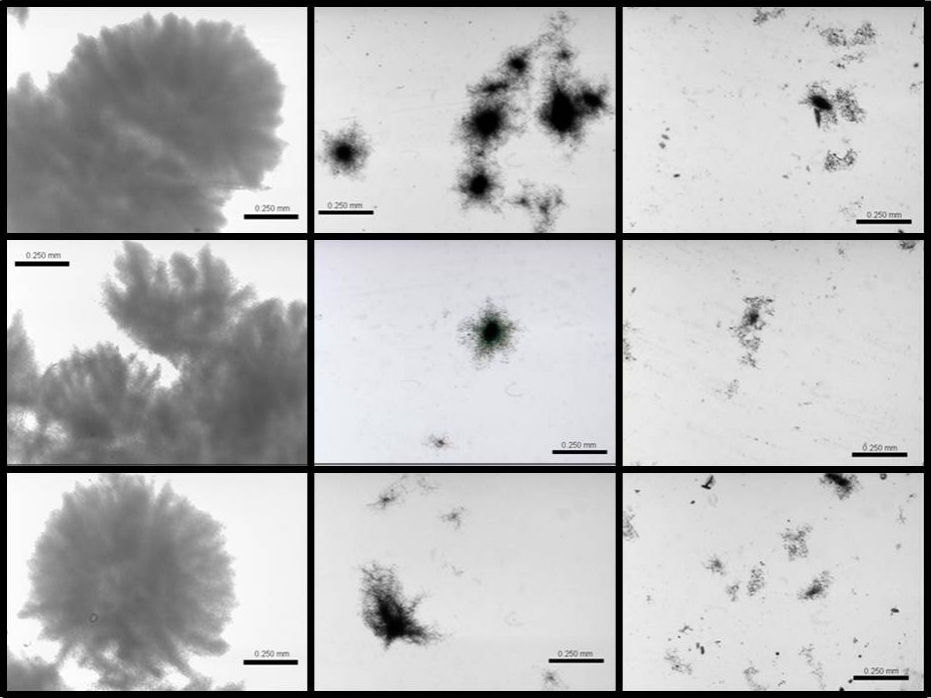
**Representative mycelial morphology of *S. lividans *cultured A. Conventional Normal (NF); B. Baffled (BF); and C. Coiled shake Flasks (CF). (Bar indicates 250 μm, 4X magnification)**.

In order to determine the characteristics of mycelial aggregation, samples were taken at 60 h from each culture and fixed to avoid the loss of morphology. These samples were analyzed by microscopy (Figure 3), and the average diameter, area, perimeter, and roundness of each aggregate was determined (Table 1). The largest and roundest pellets, with diameters up to 1.0 mm, were observed in conventional flasks; the smallest clumps, with diameters below 0.25 mm, were obtained in baffled and coiled shake flasks (Table 1, Figure 3). These morphological differences might be attributed to a greater power input/oxygen transfer rate in coiled and baffled shake flasks than in conventional ones. Based on the equations reported by Büchs et al. [39,40], the volumetric power input (P/V) for conventional shake flasks (250 ml, filled with 50 ml), at 150 rpm should be around 0.22 W/L, but the P/V for baffled shake flasks will be more than five times higher, calculated under similar conditions [39-41]. Currently, there are no equations reported for P/V in coiled shake flasks. However, since the size on the aggregates is not significantly different in coiled and in baffled flasks, then it would be expected that the power input in coiled flasks should be similar than in baffled flasks. It is worth mentioning that the power input supplied to shake flasks is directly related to the oxygen transfer rate (OTR) [42,43]. Then, it can be assumed that the OTR in baffled and coiled flasks is significantly higher than the OTR in conventional shake flasks. Based on the morphology data, between coiled and baffled shake flasks (Table 1), it can be assumed that shear and oxygenation conditions are higher in coiled and baffled flasks than in conventional normal flasks.

**Table 1:** Morphological differences in cultures carried out in conventional Erlenmeyer normal (NF), baffled (BF), and coiled shake flasks (CF). Data are presented as average ± standard deviation of image analysis of at least 100 clumps, mycelia or pellets for each sample, and at least 3 samples were analyzed for each flask. Values with the same superscript (a,b or c) are not significantly different by Tukey HSD Test (p = 0.01).

Shake flask	Area (mm^2^)	Diameter (mm)	Perimeter (mm)	Roundness (-)
Conventional	2.11 ± 1.22	1.57 ± 0.41	6.15 ± 2.32	1.51 ± 0.49
Baffled	0.04 ± 0.02 ^a^	0.23 ± 0.06 ^b^	1.01 ± 0.35 ^c^	2.11 ± 0.67
Coiled	0.02 ± 0.01 ^a^	0.16 ± 0.05 ^b^	0.77 ± 0.30 ^c^	2.66 ± 0.98

On the other hand, the larger pellets obtained in conventional flasks have a more rounded shape than the smallest clumps obtained in the other geometries. Although, no porosity measurements were carried out, the pellets can be seen to be very compact, which could bear the thought of nutritional and/or diffusional limitations within them [44], but not critical enough to affect bacterial growth. Manteca et al. [45] observed that the centre of mycelium pellets can contain inactive cells, due to a rapid consumption of substrates (nutrients and/or oxygen) within the kernel of the pellets. However, Yun et al. [38] reported that mass transfer concerns were negligible in *S. lividans *growth, when pellets reach diameters around 2 mm, at least in terms of oxygen transfer [38,46]. Moreover, in *S. lividans *larger pellets some diffusional limitations might affect the internalization of the inducer, thus reducing recombinant protein production [38], total protein production [38] or the mechanisms involved in post-translational modifications of recombinant proteins [37,47].

### Identification and quantification of rAPA produced in three flasks geometries

**Figure 4:**
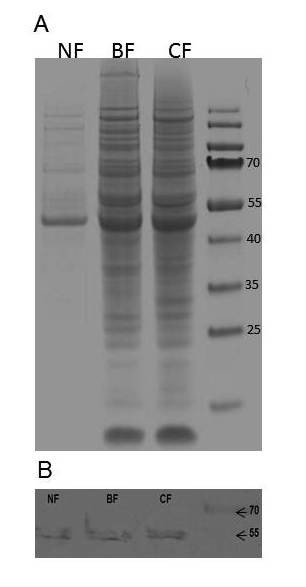
**A. SDS-PAGE of total secreted proteins of Conventional Normal (NF), Baffled (BF) and Coiled shake Flasks (CF). B. Western blot of rAPA production by *S. lividans *in the shake flasks used**.

The total secreted proteins were analyzed by SDS-PAGE and densitometry, demonstrating that rAPA was produced after induction in all flasks but in different concentrations (Figure 4A). Densitometry analysis shows a higher production of total protein in coiled and in baffled flasks than in conventional flasks (Figure 4A) and confirmed using the BCA Protein Assay Kit obtaining 4.02 ± 0.08 g/L in normal flasks and 7.44 ± 0.15 g/L in baffled and coiled flasks. Moreover, two characteristic bands were observed at 45 and 47 kDa, and recognized by mAb 6A3 in Western blots (Figure 4B) as previously reported in *S. lividans *cultures [15,24] and in *M. tuberculosis *secreted proteins [12,14,48]. Densitometry analysis of SDS-PAGE shows 3 times higher production of rAPA in coiled and in baffled flasks than in conventional flasks (Figure 4A). Those results correlated with the aggregation morphology, showing higher production in smaller pellets and lower production in the largest ones. Considering that there are no changes in the final biomass concentration, differences in total protein and in rAPA production can be attributed to the morphological variations, most probably caused by the dissimilar culture conditions inside the flasks, *i.e. *hydrodynamic and oxygen and nutrients transfer conditions.

### Analysis of O-mannosylation in rAPA

**Figure 5:**
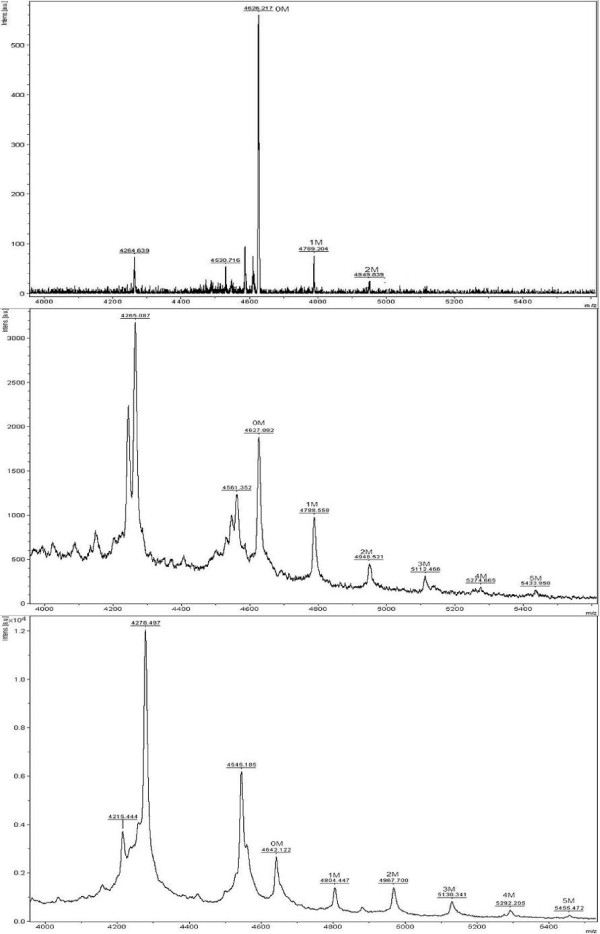
**MALDI-TOF analysis for the P8 C-terminal rAPA peptide generated by LysC digestion and obtained in A. Conventional Normal (NF); B. Baffled (BF); and C. Coiled shake Flasks (CF)**. Numbers above each peak mean the number of mannose units linked to the peptide. At least three MALDI-TOF analyses were done at the end of each independent culture.

*O*-linked glycans at the C-terminal region of rAPA were characterized. rAPA was purified and digested with LysC, producing at least eight peptides (Table 2). Native APA from *M. tuberculosis *shows in the N-terminal region 0 to 5 mannose residues that correspond to peptide 1, and in the C-terminal region 0 to 3 mannose residues located in peptide 8 [10,11]. All peptides were analyzed by MALDI-TOF with special focus on P1 and P8. In Figure 5, a typical MALDI-TOF analysis for P8 for each shake flask is shown. In all cases P1 was detected, but only in its non glycosylated form, since peaks corresponding to the mannosylated P1 peptide could not be clearly detected. In the case of coiled and baffled shake flasks, P8 shows five mannose units attached (Figure 5B, and 5C) with a typical difference between each observed peak of 163 Da corresponding to a single mannose residues. However, in conventional flasks only two mannose units were found attached to the P8 peptide (Figure 5A). In contrast, Lara et al. [15] reported four mannose units in the C-terminal peptide digested by Glu-C in rAPA from *S. lividans. *However, *S. lividans *was cultured in 1.0 L conventional flask (filled with 250 mL of modified LB medium, 34% of sucrose). This variation could be due to different shear and/or oxygenation conditions in the 1.0 L conventional flask if compared with those conditions presented in 250 mL conventional Erlenmeyer flask.

**Table 2:** Theoretical peptides generated by LysC digestion of rAPA from *S. lividans*.

Fragment	Cutting Position	Amino acid sequence	Peptide length	Mass (Da)
**P1**	**106**	**DPEPAPPVPTTAASPPSTAA APPAPATPVAPPPPAAANTP NAQPGDPNAAPPPADPNAP PPVIAPNAPQPVRIDNPVGG FSFALPAGWVESDAAHFDYG SALLSK**	**106**	**10310.439**
**P2**	**134**	**TTGDPPFPGQPPPVANDTRI VLGRLDQK**	**28**	**2987.365**
**P3**	**145**	**LYASAEATDSK**	**11**	**1155.227**
**P4**	**188**	**AAARLGSDMGEFYMPYPGTR INQETVSLDANGVSGSASYY EVK**	**43**	**4604.056**
**P5**	**194**	**FSDPSK**	**6**	**679.728**
**P6**	**234**	**PNGQIWTGVIGSPAANAPDA GPPQRWFVVWLGTANNPVDK**	**40**	**4199.698**
**P7**	**238**	**GAAK**	**4**	**345.399**
**P8**	**286**	**ALAESIRPLVAPPPAPAPAP AEPAPAPAPAGEVAPTPTTP TPQRTLPA**	**48**	**4624.317**

Data from Figure 5 demonstrate that culture conditions (simulated by three different flasks geometries) can affect the *O*-mannosylation of rAPA produced in *S. lividans*. At least two different degrees of rAPA *O*-mannosylation can be obtained, but both are different from that reported by Lara et al. [15]. Putting together all the results, it can be assumed that under other culture conditions, other glycoforms might be obtained in *S. lividans *or in *M. tuberculosis*, respectively.

Those *O*-mannosylation particularities suggests to play an important function in antigenic roles, because changes in glycosylation patterns of rAPA, produced in different hosts, significantly affect the ability to stimulate T lymphocytes *in vivo *[14,48]. It will be interesting to evaluate the different *O*-glycoforms produced in the same host (*S. lividans*), using different culture conditions (either in shake flasks or in bioreactors), and compare them with the native protein immune response.

## Conclusions

The relationship between shear and morphology in filamentous bacteria has been widely studied. In this work, said relationship has been corroborated for *S. lividans *producing a recombinant glycoprotein. In addition changes in rAPA production and the degree of *O*-glycosylation, both related to morphology, were observed. The different conditions of shear and mass transfer provided by the three shake flask geometries resulted in changes in aggregation morphology. Although, there were significant roundness morphological differences between baffled and coiled shake flasks, but not differences in diameter, said differences were not sufficient to affect the production of rAPA and the degree of C-terminal *O*-mannosylation. However, the morphology of larger pellets obtained in conventional shake flasks causes a significant decrease in rAPA production and C-terminal peptide *O*-mannosylation.

To our knowledge, this is the first report indicating how culture conditions can affect the *O*-glycosylation degree in recombinant glycoproteins produced in prokaryotes. Here, a process to obtain at least two different degrees of *O*-mannosylation, using three different flask geometries, was described. A better understanding of how hydrodynamic forces and oxygen/nutrient transfer inside *S. lividans *aggregates modify the ability to *O*-glycosylate recombinant proteins can be useful in further production processes, with the possibility to manipulate the immunogenic properties. Then, it would be interesting to separately determine and quantify the effect of shear stress and oxygen transfer rates in shake flasks, wherein dissolved oxygen is not controlled, in order to have evidences that can be useful to scale-up the production to a bioreactor.

## Methods

### Microorganism and culture conditions

Wild type *S. lividans *66 strain 1326 [32] was transformed with plasmid pIJ6021MT-45 carrying the *apa *gene under a thiostrepton-inducible promoter, and conferring resistance to kanamycin [15]. A master stock of 30 mL of spores in 20% glycerol was obtained, divided into aliquots, and kept at -20°C until use. To start cultures spores were pregerminated in 2XYT medium for 6 h at 37°C and 150 rpm [15]; the germinated spores were then washed and sufficient spores were inoculated in order to obtain an optical density of 0.025 at 600 nm (measured in a Beckman DU730 spectrophotometer). Three different shake flask geometries were tested (250 ml, filled with 50 ml of medium): Coiled Flask (CF), Baffled Flask (BF), and conventional Erlenyemer Normal Flask (NF), as is shown in Figure 1. Coiled flasks consist of conventional normal flasks with an inserted 30 cm stainless steel spring (1.3 cm diameter, 19 sw gauge) as previously described [32]. Culture medium was Luria-Bertani's medium with kanamycin (50 μg/mL) modified by addition of 34% w/v sucrose [15]. All cultures were carried out at 30°C and 150 rpm for 60 h, with addition of the inducer (thiostrepton 10 μg/mL) at 16 h of culture. Two flasks were removed at each kinetic data point for biomass, protein and image analysis.

### Analytical determinations

Biomass was evaluated by dry weight; 10 mL of culture were filtered through a 0.45 μm pore size membrane (Millipore), and washed once with one volume of distilled water. The mycelium obtained was dried for 24 h in an oven at 55°C, then placed for 2 h in a desiccator, and weighed afterwards.

### Image analysis procedures

A sample of 20 μL of culture broth was fixed using a formaline solution (10% v/v) in order to avoid the loss of the actual morphology in fresh samples. This sample was placed on a slide, and carefully covered with a cover slip. At least 100 clumps, mycelia or pellets were analyzed for each sample, and at least 3 samples were analyzed for each flask. 2D images were obtained by an image analysis system consisting of a CCD camera (Nikon Color, KP-D50) coupled to a microscope (Nikon Optiphot-2), or a CCD camera (Nikon KP-160) mounted to a stereomicroscope (Olympus SZ40), and a PC with the image analysis software (Image Pro Plus 4.1, Media Cybernetics, Silver Spring, MD) installed in it [49]. The software reported the average diameter and the roundness to characterize the size of aggregates, as the main results. Average diameter is defined as the diameter of a perfect circle, having the same area as the object to be measured. Roundness describes the deviation of mycelial particles from a true circle. In large aggregates (from conventional shake flasks), images were first converted to monochrome and then these grayscale images were processed using a special algorithm for aggregates [50]. One-way ANOVA for independent and samples and pair-wise comparisons using Tukey HSD (Test for Post-ANOVA) were carried out in order to assess the morphological statistical differences between shake flask geometries. Analysis were done using Excel^® ^(2007) and the " VassarStats: Website for Statistical Computation" available on-line of Vassar College, NY-USA (http://faculty.vassar.edu/lowry/anova1u.html).

### Purification of rAPA

All cultures were centrifuged at 10,000 × *g *and the supernatant was filtered through a 0.45 μm membrane (Millipore). Ammonium sulfate was added to the culture filtrate to 75% saturation and the precipitated proteins were recovered by centrifugation, suspended in PBS (Phosphate Buffered Saline; 10 mM Na_2_HPO_4_, 2 mM KH_2_PO_4_) buffer, dialyzed against distilled water overnight, subsequently against an acetate buffer (Acetic acid 0.2 M, Sodium acetate 0.2 mM, pH 5.0) for 24 h, and finally against ConA binding buffer (Sodium acetate 0.1 M, NaCl 1.0 M, Mn_2_-4H_2_O 1 mM, Sodium Azide 0.01% and CaCl_2_-2 H_2_O 1 mM) for 24 h. The ConA-binding protein (47-kDa protein) was separated by affinity chromatography on ConA-Sepharose column (Concanavalin A from *Canavalia ensiformis*, Sigma-Aldrich), and eluted with the ConA binding buffer with α methyl-mannopyranoside 0.05 M.

### Protein identification and quantification

Total protein was determined using the Thermo Scientific Pierce BCA Protein Assay Kit (Thermo-Pierce) from supernatant. Electrophoresis in 10% polyacrylamide gels containing SDS and subsequent immunoblotting procedures were carried out as previously described [15]. For Western blots, 100 μg of total protein in SDS-PAGE (10%) were transferred to polyvinylidine difluoride membranes (Millipore). Nonspecific binding was blocked with 5% (w/v) skim milk in PBS containing Tween 20 (0.05% v/v) for one hour. After that, membranes were washed with PBS Tween 0.05%. The primary antibody mAb6A3 was added in dilution 1:500 and incubated overnight at 4°C [15]. After three washes with PBS-Tween 20, the membranes were incubated with diluted peroxidase-conjugated anti-mouse IgG 1:1000 (Sigma-Aldrich). After incubation, the blots were stained for peroxidase activity by adding 3,3-diaminobenzidine (Sigma-Aldrich) and hydrogen peroxide (Sigma-Aldrich) in PBS and washed with tap water.

### Protein digestion

The 45 and 47 kDa bands were excised and washed with water, distained with ammonium bicarbonate buffer (100 mM) with 50% methanol, dehydrated with acetonitrile (100%), and rehydrated with ammonium bicarbonate buffer (25 mM) containing enzyme Lys-C (Roche) in dilution 1:1000. Digests were incubated overnight at 37°C, 20 μL of Trifluoroacetic acid (TFA, 0.1%) were added to stop the reaction, and concentrated in a SpeedVac concentrator (Savant-Thermo) down to a volume of 20 μL.

### MALDI-TOF Analysis

Masses were determined in a Bruker Microflex matrix-assisted laser desorption ionization time-of-flight instrument (Bruker Daltonics GmbH, Leipzig, Germany) equipped with a 20-Hz nitrogen laser at l = 337 nm. Spectra were recorded in reflector and/or linear positive mode for the mass range of 3800 - 6000 Da. 1.0 mL of sample solution was mixed with 5 mL of 30% Acetonitrile, 70% Water, 0.1% TFA, and saturated with a-cyano-4-hydroxycinnamic acid or sinapinic acid. Then, 1.0 μL of this solution was deposited onto the MALDI target and allowed to dry at room temperature. At least three MALDI-TOF analyses were done at the end of each independent culture.

## Competing interests

The authors declare that they have no competing interests.

## Authors' contributions

MATR, NAVC, LSG and CE conceived, supervised and coordinated this study. RAGS participated in the experimental design, carried all cultures. LECD and RAGS carried out the transformations and participated in master stock elaboration. JAMS and RAGS participated in protein purification and identification. RAGS and NAVC carried out the protein digestion and the interpretation of MALDI-TOF analysis. RAGS, MATR and NAVC drafted the manuscript, CE and LSG revised it critically and amended the manuscript. All authors read and approved the final manuscript.
